# Corrigendum to “Risk Factors for Fracture in Diabetes: The Canadian Multicentre Osteoporosis Study”

**DOI:** 10.1155/2017/3689474

**Published:** 2017-07-12

**Authors:** Lisa-Ann Fraser, Alexandra Papaioannou, Jonathan D. Adachi, Jinhui Ma, Lehana Thabane

**Affiliations:** ^1^Department of Medicine, University of Western Ontario, 339 Windermere Road, London, ON, Canada N6A 5A5; ^2^Division of Endocrinology and Metabolism, St. Joseph's Hospital, 268 Grosvenor Street, London, ON, Canada N6A 4V2; ^3^Department of Epidemiology and Biostatistics, McMaster University, 1280 Main Street West, Hamilton, ON, Canada L8S 4K1; ^4^Department of Medicine, McMaster University, 1280 Main Street West, Hamilton, ON, Canada L8S 4K1; ^5^Centre for Evaluation of Medicines, St. Joseph's Healthcare, 50 Charlton Avenue East, Hamilton, ON, Canada L8N 4A6

In the article titled “Risk Factors for Fracture in Diabetes: The Canadian Multicentre Osteoporosis Study” [1], there was an error regarding the FRAX® tool, which should be clarified as follows.

The article notes: “The WHO fracture risk assessment tool (FRAX), a commonly used fracture prediction tool, has been found to underestimate fracture risk in the diabetic population [10].” However, the World Health Organization (WHO) did not develop, test, or endorse the FRAX tool or its recommendations [2]. The metabolic bone disease unit at the University of Sheffield that developed FRAX was a WHO Collaborating Centre from 1991 to 2010, but treatment guidelines must undergo a formal process before they can be endorsed by the WHO.
